# The Effect of Short-Term Exposure to Cadmium on the Expression of Vascular Endothelial Barrier Antigen in the Developing Rat Forebrain and Cerebellum: A Computerized Quantitative Immunofluorescent Study

**DOI:** 10.7759/cureus.23848

**Published:** 2022-04-05

**Authors:** Michael O Ibiwoye, Emily A Snyder, James Lyons, Audrey A Vasauskas, Mark J Hernandez, Arthur R Summerlin, James D Foster

**Affiliations:** 1 Anatomy and Molecular Medicine, Alabama College of Osteopathic Medicine, Dothan, USA; 2 Research, Alabama College of Osteopathic Medicine, Dothan, USA; 3 Department Clinical Sciences, Alabama College of Osteopathic Medicine, Dothan, USA; 4 Institutional Effectiveness, Alabama College of Osteopathic Medicine, Dothan, USA; 5 Pathology, Southeast Health Medical Center, Dothan, USA

**Keywords:** blood–brain barrier, endothelial barrier antigen, automated digital image analysis, cavalieri principle, stereology, cadmium neurotoxicity, rat

## Abstract

Clinical and laboratory studies have shown that environmental exposure to cadmium produces damage to several organs, including bones, lungs, and kidneys. The involvement of cadmium in central nervous system (CNS) disorders has also been widely reported, but the precise pathophysiological mechanism is not yet fully understood. Children who were exposed to cadmium during pregnancy are known to suffer from developmental delays, learning difficulties, attention deficit hyperactivity disorder (ADHD), and other cognitive and neurobehavioral deficits. Results from numerous studies suggest that dysfunction of the blood-brain barrier (BBB) structures is an important step in the neurotoxicity of cadmium. A rat-specific BBB marker protein, the endothelial barrier antigen (EBA), has been previously isolated and classified by Sternberger and others. The mouse IgG1 clone, anti-endothelial barrier antigen (anti-EBA), detects a protein triplet (23.5kDa, 25 kDa, and 30kDa) localized to the luminal surface of central and peripheral nervous system (CNS and PNS) vascular endothelial cells with selective permeability barrier functions. This marker has been widely used for characterizing BBB alterations under demyelinating, inflammatory, and other CNS pathologies. Many studies have been published using the rat model system for studying the neurotoxic effect of acute and chronic exposure to cadmium.

We applied the indirect immunofluorescent techniques using the anti-EBA antibody in conjunction with the Olympus cellSens computerized image analysis to detect and quantify the surface areas of BBB-competent microvessel profiles in paraformaldehyde-fixed, paraffin-embedded brains of term-delivered young rats after intraperitoneal injection of a single dose of cadmium chloride. We detected a statistically significant reduction in EBA-positive microvessel surface areas in the forebrain (t = 5.86, df = 1789, p-value < 0.001) and cerebellum (t=73.40, df=1337, p < 0.001) of cadmium-treated rats compared to the normal controls. Thus, this study supports the hypothesis that the EBA is a sensitive and measurable indicator for quantitative assessment of the impact of cadmium exposure in the developing rat brain.

## Introduction

Cadmium is a heavy metal typically found in association with zinc, lead, and copper ores as natural components of the earth’s crust, and its harmful effects are well known worldwide. Cadmium is known to cause damage to many organs and tissues, including the testes, kidneys, bones, lungs, and brain [1-10]. The metal is found in at least 1,014 of the 1,669 most hazardous sites identified and designated as National Priorities List (NPL) by the Environmental Protection Agency (EPA), indicating the public health significance of cadmium as an environmental pollutant [11-13]. As further evidence of its hazardous potential, the International Agency for Research on Cancer (IARC) has classified Cd as a Group 1 carcinogen in humans [11]. Cadmium typically contaminates the drinking water, air, and soil via a wide range of anthropogenic activities such as industrial production of rechargeable batteries, smelting, electroplating, phosphate fertilizers, and high-volume waste disposal by incineration. Through these activities, the metal readily enters the human food chain, rendering it a worldwide food and environmental contaminant [14-18]. A review of the literature indicates that tobacco leaves accumulate significant amounts of cadmium from the soil and are directly associated with exacerbation of respiratory pathologies, including chronic obstructive pulmonary disease (COPD) and emphysema, especially in heavy cigarette smokers [11,19-23]. Reports have also been published showing that cadmium can be released into the air, soil, and water through natural processes such as soil and rock erosion, wildfires, and volcanic eruptions [15,18,24,25]. Occupational and non-occupational exposure potential to Cd is very high, and studies have shown that maternal exposure during pregnancy results in children suffering from abnormalities, including growth retardation, learning disorders, neurobehavioral and other cognitive disabilities postnatally [12,26-31,32-38]. According to the Agency for Toxic Substances and Disease Registry [11], dietary ingestion constitutes the highest source of cadmium exposure for non-smoking adults and children in the United States. Among United States residents, the daily ingestion of cadmium in non-smoking adult males and females has been estimated at 0.35 and 0.30 μg Cd/kg/day, respectively [11]. The adverse impact of cadmium is further worsened by its slow elimination and long biological half-life in the body [39]. The mechanism by which Cd produces damage to the brain has been a subject of extensive investigations but is not yet completely elucidated. It has been hypothesized that Cd neurotoxicity is due to its key role in oxidative stress-induced morphological and functional perturbation at the cellular level, including blood-brain barrier (BBB) structures [7,40-47].

The electron microscopic studies by Reese and Karnovsky [48] and by Brightman et al. [49] have shown that the mammalian blood-brain barrier (BBB) location is at the brain microvascular endothelial cells (BECs) [50]. Until recently, it was impossible to selectively identify or observe the individual cells of the BBB due to the lack of a specific marker for the functionally and morphologically unique microvessels [47,51-55]. During postnatal development of the vertebrate mammalian brain, including rats and humans, in response to brain-derived factors, the BECs acquire barrier characteristics (barrier genesis), including tight junction-associated proteins phenotype [56-63]. The identification and localization of markers within single cells and in tissues by immunohistochemistry with specific antibodies have greatly facilitated in-depth studies of the underlying molecular mechanisms of diseases, including BBB alterations under pathological conditions [5,40,56-58,63-71]. A microvascular endothelial marker, specific to rat BBB, the endothelial barrier antigen (EBA), has been identified and classified by Sternberger et al. [72] based on the lymphocyte fusion technique of Köhler and Milstein [73], using rat whole brain homogenate as immunogen. The monoclonal antibody, anti-endothelial barrier antigen (anti-EBA), reacts exclusively with neurovascular endothelia with permeability barrier functions. It is unreactive with non-endothelial cells in or outside of the nervous system. Additionally, fenestrated microvessels of peripheral body organs such as the heart, liver, and kidney, and microvessels of the brain circumventricular organs (CVOs), do not display immunoreactivity with anti-EBA. Previous studies have utilized EBA expression as a valid model for evaluating BBB alterations under normal and pathological conditions [74-80]. In a previous study using Sprague-Dawley rats, Ghabriel et al. [43] reported a decrease in EBA immunoreactivity and extensive extravasation into the brain parenchyma of endogenous albumin and intravenously administered horseradish peroxidase (HRP) after a single parenteral injection of anti-EBA monoclonal antibody. This observation suggests an inverse correlation between BBB disruption and the brain's microvascular EBA content. More recently, Pelz et al. [81] demonstrated a similar inverse correlation between EBA reactivity and BBB disruption in focal cerebral ischemia induced by an embolic stroke model in Wistar rats. In an earlier study utilizing Lewis rats with induced experimental allergic encephalomyelitis (EAE), a rodent analogue of human multiple sclerosis (MS), Sternberger et al. [82] detected a complete loss of immunoreactivity with anti-EBA in brain microvessels surrounded by inflammatory cells within the brain lesions. In the same study, it was also reported that BECs in rats that had recovered from EAE reacted with the antibody, while microvessels in brain areas with persistent inflammation remained EBA-negative, suggesting that EBA reappeared during recovery from EAE). The data from several studies have shown that in the developing rat and human brain, the immature, nascent BBB is potentially more sensitive to the effects of exogenously or environmentally induced pathogenic agents, including cadmium-induced neurotoxicity [40,83]. Despite many reports on BBB changes using EBA as an indicator [43,81-82], the impact of cadmium exposure on possible alterations in EBA as an indicator has not been widely studied. The results from previous work by other investigators have revealed that prenatal, newborn, and adult rats are valid model systems for studying the neurotoxicity of parenterally or orally administered cadmium in the setting of both acute and chronic exposures to the metal [84-88].

The goal of the present study was to evaluate alterations in the postnatal expression of the EBA as a rat-specific BBB indicator by objectively and accurately determining the surface areas of EBA-positive microvessels in the forebrain and cerebellum in young rats. We applied the indirect immunofluorescence protocols with anti-EBA and an Olympus cellSens automated digital imaging software suite to determine the surface areas of BBB-competent microvessels within standardized microscopic fields, following a short duration exposure to cadmium by intraperitoneal injection of neonate rats.

## Materials and methods

Animals and brain tissue preparation

**Figure 1:**
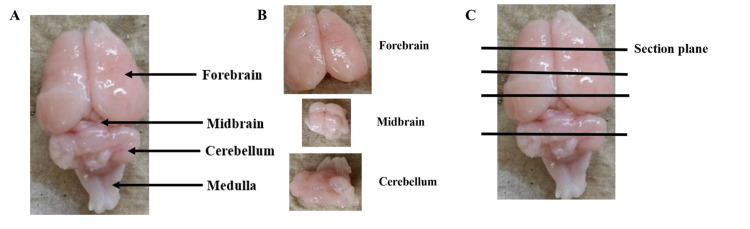
Isolation and sectioning of rat brain regions. Representative rat brain isolation and sectioning orientation, (A) Whole-brain dorsal view. (B) Detached forebrain, midbrain, and cerebellum. (C) Coronal plane orientation for tissue block preparation and microtome thin (8 µm) sectioning.

Two equal groups of 40-day-old healthy male Sprague-Dawley rats weighing 150-200 g (Charles River Laboratories Inc., USA) were utilized for this study. Male rats were used based on the data from previous studies showing that exposure of female rats to cadmium caused suppression of the immune system, including altered responses of the spleen cells [89]. Furthermore, we used animal cohorts matched for age, sex, and strain to ensure reliable between-group comparisons, given the previously published data suggesting a differential expression of the EBA within different rat brain regions [75, 89-91]. The present study was performed under stringent adherence to the Institutional Animal Care and Use Committee (IACUC) and the Institutional Review Board (IRB) regulations of the Alabama State University and the Alabama College of Osteopathic Medicine (ACOM), respectively. Both the experimental and control animals, 10 animals per group, were housed two per cage and allowed free access to standard rat chow and drinking water throughout the experiment. After 10 days of adaptation to the laboratory environment, a group of 10 animals was parenterally (intraperitoneally, IP) injected with a single dose of cadmium chloride (Sigma-Aldrich, St. Louis, Missouri, USA) at a dose of 4 mg/kg body weight dissolved in isotonic saline [75]. This cadmium dose was chosen based on previous studies by other authors on the toxic effects of short-term exposure to cadmium in the brain and peripheral organs of young rats [13,84,92]. The second group of 10 rats received IP isotonic saline injection each and was used as the normal control group. Three days post-injection, all rats were sacrificed by exposure to increasing concentration of carbon dioxide followed by cervical dislocation. After removal, each brain was transferred into a Petri dish with a unique identification code and placed on wet ice at 4°C. The cadmium administration and all subsequent dissection steps were carried out in a laminar flow hood. The brain was kept wet throughout the dissection with an intermittent application of a jet of cold 4% paraformaldehyde in phosphate-buffered saline (PBS, pH 7.4) to facilitate dissection and preserve tissue antigen. The forebrain was rapidly detached from the midbrain and cerebellum by making a transverse incision at a level corresponding to the rostral border of the superior colliculus, as previously described [75]. Next, the cerebellum was separated from the midbrain by cutting transversely through the brainstem at the level corresponding to the caudal border of the inferior colliculus. Finally, the cerebellum was detached from the brainstem by gently retracting it upward and cutting transversely through the rostral medulla. The isolated forebrain and cerebellum from each rat were then cut in a coronal plane into blocks and immersion-fixed overnight at 4°C in 120 ml specimen bottles precoded with the rat numbers containing 4% paraformaldehyde in phosphate-buffered saline (PBS, pH 7.4). The forebrain and cerebellum were each cut into four and two coronal slices, respectively (Figure 1). To ensure consistency of between-group comparison of similar brain regions, every second and third block from each forebrain starting from the rostral end and two blocks from the cerebellum of experimental and control rats were placed in cassettes with original rat identification codes and processed to paraffin embedding overnight in a vacuum infiltration tissue processor (Tissue-Tek, Sakura Finetek, Torrance, CA, USA). The blocks were embedded in a coronal plane and sectioned serially at 8 μm using a rotary microtome (Microm M355S, ThermoFisher, USA). Every fifth section from the paraffin-embedded blocks was mounted on Superfrost glass slides (Fisher Scientific, Pittsburgh, PA, USA) labelled with the original identification codes and immunofluorescently stained with anti-EBA for the computerized image analysis as described below. To confirm tissue specificity of an anti-EBA monoclonal antibody, small pieces (~ 3cm^3^) were resected from the kidney and processed to paraffin embedding as above, and thin sections (8 μm) were cut. In some cases, some brains were sectioned in a para-sagittal plane, revealing the fourth ventricular cavity, ependymal epithelial lining, and the choroid plexus to examine the vascular endothelium-specificity of EBA. Additionally, thin sections from the brain and peripheral organs were stained with monoclonal antibody CD105 (endoglin) as a general vascular endothelial marker for distribution comparison with anti-EBA as a rat BBB-specific vascular endothelial marker.

Preparation for immunofluorescent detection of EBA-positive microvessels

To visualize the EBA immunoreactivity in normal control and cadmium-treated paraffin-embedded brains, we used affinity-purified mouse anti-rat BBB monoclonal antibody (IgM, clone SMI-71, Biolegend, Inc., San Diego, USA). The indirect immunofluorescent detection was performed as follows: the sections were dewaxed for 5 minutes in each of three changes of xylene followed by rehydration for 5 minutes in each of two changes of descending grades of absolute, 95%, 70% and 50% ethanol; immersed for 5 minutes in each of two changes of deionized water and two changes of PBS; pretreated with trypsin antigen retrieval solution (ab970 - ABCAM Inc., Cambridge, MA, USA) for 10 minutes in an Isotemp incubator (Thermo Fisher Scientific, Waltham, MA USA) maintained at 37°C for optimal EBA detection; non-specific binding was blocked by treating sections for 1 hour in a moist slide chamber at room temperature with animal-free diluent and blocker reagent (Cat# SP-5035-100; Vector Laboratories, Inc. Burlingame, CA, USA); excess blocking solution was gently tapped off followed by overnight incubation (4°C) with affinity-purified mouse anti-rat EBA monoclonal antibody SMI-71 (IgM) (cat #836804; Biolegend, San Diego, USA) diluted 1:1000 in animal-free diluent; washed for 5 minutes in each of two changes of PBS (pH 7.4); incubated at room temperature for 1 hour with donkey anti-mouse fluorescein isothiocyanate (FITC)-conjugate (Jackson ImmunoResearch Laboratories, USA) at a dilution of 1:400 using animal-free diluent; finally, the immunostained slides were washed in two changes of PBS as above. Labeled sections were mounted in a VectaShield vibrance antifade mounting medium containing 4′,6-diamidino-2-phenylindole (DAPI) as nuclear counterstain (cat #H-1800; Vector Laboratories Inc., Burlingame, CA, USA). Negative control tissue sections were treated with the same immunohistochemical reagents under identical conditions, but the primary antibodies were skipped. EBA-positive microvessels appeared as green, fluorescent profiles, while the nuclei appeared as blue structures under the Olympus BX63 epifluorescence microscope.

Preparation for heat-activated epitope recovery and immunofluorescent detection of CD105 (endoglin)

The detection of CD105 in paraffin-embedded kidney and brain sections was carried out as follows: sections were dewaxed as described above and rehydrated to PBS (pH 7.4) and tap water; the sections were subjected to 3 minutes heat-mediated antigen retrieval under constant boiling temperature and pressure in a pressure cooker containing 2 liter 100 Mm Citrate buffer (pH, 6.0) (catalogue #ab93678, ABCAM, Cambridge, Massachusetts, USA); cooled under gentle running tap water; permeabilized by washing for 5 minutes in each of two changes of 0.01%Triton X-100 (Fisher Scientific, Pittsburgh, PA, USA ) in PBS (pH 7.4); washed in two changes of PBS (pH 7.4); non-specific binding blocked by incubation in animal-free blocking/diluent reagent (Vector Laboratories, Burlingame, CA, USA) for 1 hour at room temperature to reduce non-specific binding by secondary antibody; incubated overnight (4 °C) in rabbit anti-CD105 monoclonal antibody (ABCAM, Cambridge, Massachusetts, USA) (1:50 dilution in animal-free diluent and blocker); washed in 2 changes of PBS (pH 7.4) and incubated in goat anti-rabbit FITC-conjugated secondary antibody diluted 1:400 in animal-free diluent for 1 hour at room temperature; washed twice in PBS and mounted in Vectashield vibrance medium with diamidino-2-phenylindole (DAPI) (Vector Laboratories, Burlingame, CA) as nuclear counterstain.

Negative control brain sections were treated with the same immunohistochemical reagents under identical conditions, but the primary antibody was omitted. Some rat brains were sectioned in a sagittal paramedian plane revealing portions of the pons, cerebellum, ventricular ependymal lining, and the choroid plexus were also stained with anti-EBA to serve as internal controls and for comparison with the anti-CD105 distribution. In addition, paraffin-embedded sections from the kidney, known to possess both fenestrated and continuous capillaries lacking a permeability barrier function, were immunostained with the anti-CD105 for comparison with anti-EBA specificity.

Quantitative computerized image analysis

Overall, two blocks from the forebrain and two blocks from the cerebellum were used per experimental and control rat, and five fields of view (FOVs) from each of eight thin sections were analyzed per block for this study. Before surface area measurement, the original rat identification codes on the slides were covered with tape, and each slide was randomly assigned a number to eliminate observer bias in the assessment. From a random point within each tissue section, every second FOV in equidistant series of fields was examined. All FOVs were evaluated per slide and each slide was assessed thrice by the same observer. Computerized image analysis was carried out as follows: the immunofluorescently labeled sections were examined, and images were acquired with an Olympus BX63 fluorescence microscope equipped with a DP80 CCD color camera and cellSenseTM Dimension software program (Olympus, cellSens Dimension 1.17 Build 21199). The cellSens Dimension software suite incorporates a Count and Measure module that allows for global thresholding to segment immunofluorescently labeled profiles of interest from the surrounding background and measure surface areas accurately and reproducibly within the standardized region of interest (ROI). The software can also determine the number of immunofluorescently labeled profiles within the defined ROI. We used the entire field of view (FOV) in a ×40 objective lens (ROI) for the evaluation, which was equivalent to an area of 90610.43 mµ2 (0.1 mm2) that was automatically generated by the software on the computer screen. The mean surface areas of EBA-positive microvessels were measured in each field for all slides from both the cadmium-treated and control rat brains. The grand means (Mean ± SE) were computed for both groups of rats for statistical analysis.

**Figure 2:**
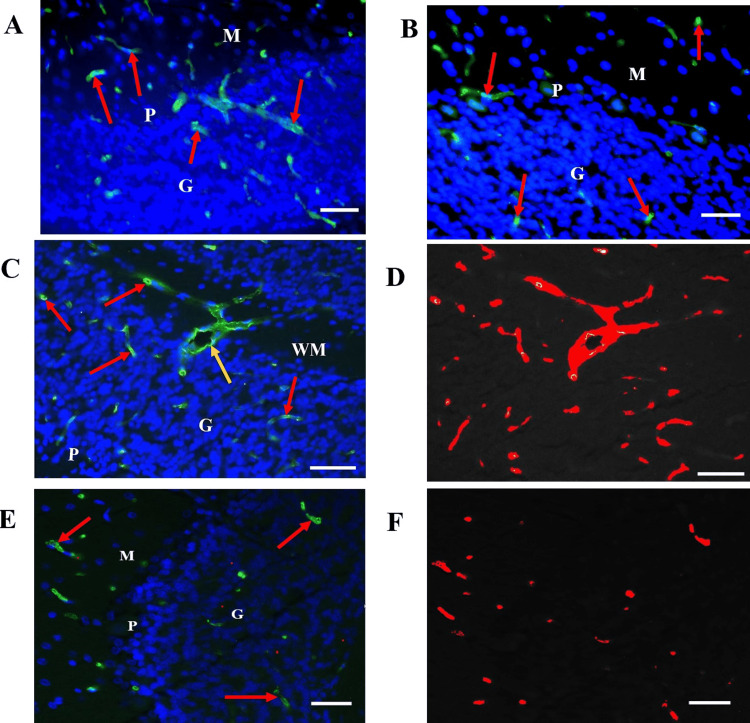
EBA-positive microvessels are reduced in the cerebellum of cadmium-treated rats. (A) EBA immunoreactivity in a control rat cerebellum showing strong labeling of microvessels by anti-EBA (red arrows). (B) Cadmium-exposed rat cerebellum showing decreased EBA-positive microvessels (red arrows). (C) to (F) Microvessel surface area measurement in normal control (C) and cadmium-treated (E) rat cerebellum showing EBA-immunopositive venules (yellow arrows) and capillaries (red arrows) and their collaterals. (D) & (F), global thresholding and segmentation of EBA-positive microvessels for surface area measurement. WM: cerebellar white matter; G: Granule cell layer; P: Purkinje cell layer. Scale bars, 20 µm.

Before surface area determination, each image was separated into its red, green, and blue (RGB) component channels, followed by the selection of the red channel and utilizing the red phase to highlight the individual immunoreactive microvessels. The global thresholding was set by using the manual threshold mode to carefully adjust the minimum and maximum intensity distribution histogram on a sliding scale until the best quality image was obtained for contrast and intensity while also ensuring that all immunoreactive microvessels were highlighted in red against a clear, black background. We determined that highlighting the EBA-reactive microvessels using the red phase instead of the green phase allowed for more precise detection and differentiation of the profiles of interest and a more complete segmentation of the background and its green endogenous autofluorescence from the brain tissue components. The same global threshold was applied for analysis of all fields subsequently examined to ensure consistency and precision of analysis (Figures 2C-2F).

The class measurement tool was used to automatically generate the number and mean surface areas of the individual EBA-positive microvessel structures within the ROI. Finally, the data generated were exported to an excel spreadsheet, and grand mean surface areas (μm2) of the EBA-positive microvessels in all microscopic fields were computed for the control and cadmium-exposed forebrain and cerebellum. These data were presented as Mean ± SE values.

Statistical analysis comparing profile grand mean surface areas of the cadmium-treated brains and normal controls was performed by Welch’s two‐sample t‐test with a confidence interval set at 95%.

## Results

Histological observations and endothelial barrier antigen immunoreactivity

**Figure 3:**
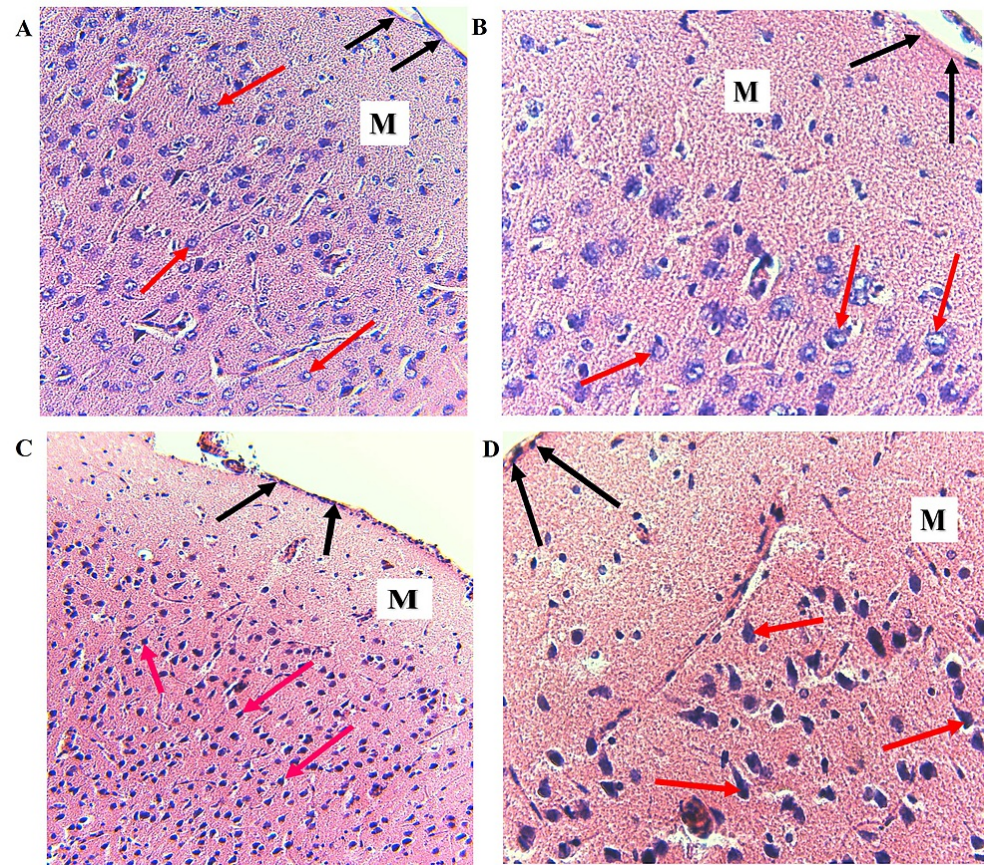
Cadmium-treated rats display evidence of neuronal and glial damage. (A) Normal forebrain, H & E. Uniformly eosinophilic, neuropil, clearly defined neuronal and glial cell bodies, open face neuronal nuclei with prominent nucleoli and visible cytoplasm (red arrows), (B) High magnification of A, showing clearly defined neuronal and glial cell bodies and nuclei (red arrows). (C) Cadmium-treated Forebrain. Dark-staining neuronal and glial nuclei with ill-defined cell bodies (red arrows). (D) High magnification of C showing dark-staining neuronal and glial nuclei and ill-defined, with dark, acidophilic cytoplasm (red arrows). M: Molecular layer. The leptomeningeal layer is indicated by black arrows. H & E: (A) and (C) (x200), (B) and (D) (x400).

**Figure 4:**
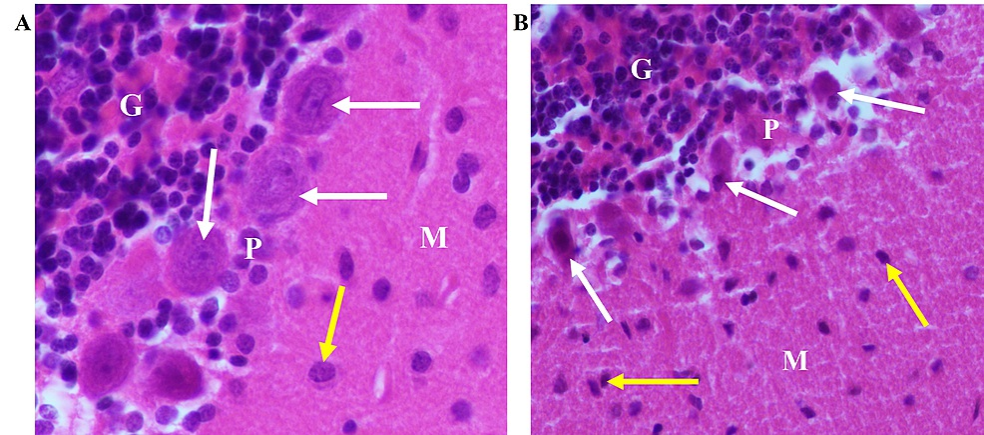
Cadmium-treated rats display abnormalities in the cerebellar cortex. (A) Normal control rat cerebellum. Well preserved, normal-appearing Purkinje neuronal (white arrows) and glial (yellow arrows) cell bodies, nuclei, and cytoplasm. (B) Cadmium-treated rat cerebellum showing hyperchromatic glial nuclei (yellow arrows) and shrunken Purkinje neurons with hyperchromatic nuclei (white arrows). M: cerebellar molecular layer; P: Purkinje cell layer; G: granule cell layer. H & E (x400).

In hematoxylin- and eosin-stained histological sections, the principal light microscopic observations were dark-staining of the neuronal and glial cell nuclei and ill-defined cell bodies that appeared to be degenerated in cadmium-treated forebrains compared to normal control brains (Figure 3). Similar sections from the cerebellum of cadmium-exposed rats exhibited hyperchromatic glial cell nuclei and irregularly shaped, Purkinje neurons, which appeared to be shrunken with strongly basophilic and homogeneous nuclei compared to the normal control cerebellum (Figure 4).

**Figure 5:**
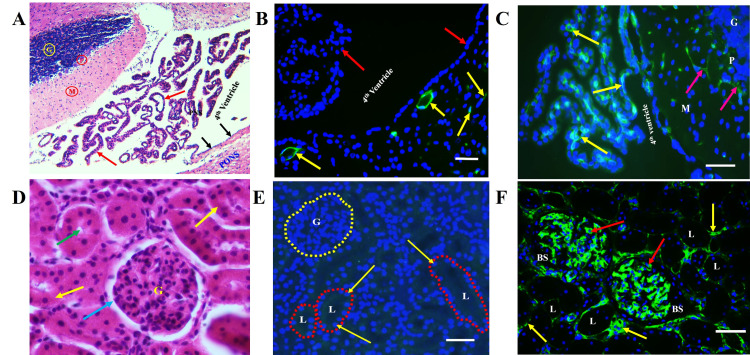
EBA is specific to brain regions. (A) Section showing the histological organization of normal rat cerebellum, adjacent fourth ventricle and choroid plexus (red arrows), and pons. The ependymal lining of the fourth ventricle is shown by black arrows; part of the cerebellum is shown in the top left corner. M: Molecular layer; P: Purkinje cell layer; G: Granule cell layer. H & E (x100). (B) Normal rat brain section stained with anti-EBA. Note positive staining of brain capillaries and venules (yellow arrows), but ventricular ependymal lining and choroid plexus epithelial cells (red arrows) are unreactive. (C) Anti-CD105 is reactive with the brain (red arrows) and choroid plexus (yellow) microvessels. (D) Kidney histological section from normal control rat. G: Glomerulus; renal tubular epithelial cells (yellow arrows); renal tubule lumen (green arrow); Bowman space (blue arrow). H & E (x400). (E) No EBA-immunoreactivity is detected in capillaries within the glomeruli (area G, surrounded by a yellow circle) or in peritubular areas (surrounded by red circles). G: Glomerulus; L: renal tubule lumen. Tubular epithelial cell nuclei are indicated by yellow arrows. (F) Section from a paraffin-embedded rat kidney showing strong labeling of the renal glomerular (red arrows) and peritubular (yellow arrows) microvasculature by anti-CD105. L: renal tubule lumen; BS: Bowman’s space. Scale bar, 20 µm

**Figure 6:**
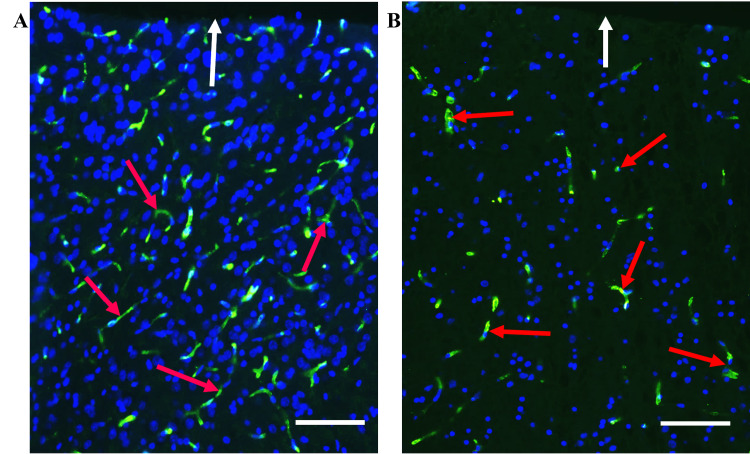
EBA-positive microvessels are reduced in the cerebral cortex of cadmium-treated rats. (A) EBA immunoreactivity in a control rat cerebral cortex showing labeling of microvessels of various sizes by anti-EBA antibody (red arrows). (B) EBA immunoreactivity in a cadmium-treated rat cerebral cortex (red arrows). The leptomeningeal layer is indicated by white arrows. Scale bars, 50 µm.

We detected immunoreactivity in microvessels in the forebrain and cerebellum of experimental and control rats (Figures 5B, 6A, 6B). By contrast, the EBA immunoreactivity was undetectable in negative control slides in which the primary antibody was skipped, or in sections from the kidney and area of the choroid plexus of the 4th ventricle, the histological organization is shown for reference (Figures 5A, 5B, 5D, 5E). A reduction in the number of EBA-positive microvessels was observed in cadmium-treated forebrain and cerebellum compared to the normal control animals (Figures 5, 6A). The anti-CD105 monoclonal antibody was detected in vascular endothelial in the sections from the kidney, brain, and choroid plexus (Figures 5C, 5F).

**Figure 7:**
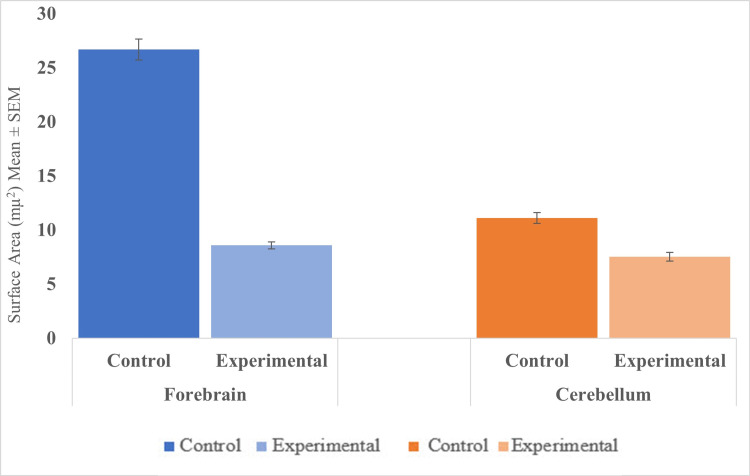
Surface areas of EBA-positive microvessels in the forebrain and cerebellum (mµ2) (Mean ± SE). Forebrain: p < 0.001; Cerebellum: p < 0.001

In normal control brains, the surface areas for EBA-positive microvessel profiles were 11.11 ± 0.5 µm^2^ (Mean ± S.E) and 26.68 ± 0.98 µm^2^ (Mean ± S.E) in the cerebellum and forebrain, respectively. In the cerebellum and forebrain of rats exposed to cadmium, the EBA-positive microvessels surface areas were 7.51 ± 0.4 µm^2^ (Mean ± S.E) and 8.58 ± 0.33 µm2 (Mean ± S.E), respectively. We found a statistically significant reduction in EBA-positive microvessel areas in the forebrain (t = 5.86, df = 1789, p-value < 0.001) and cerebellum (t=73.40, df=1337, p < 0.001) of cadmium-treated brain compared to the normal controls (Tables 1, 2, Figure 7).

**Table 1:** Mean surface areas of EBA-positive microvessels in the forebrain. N: Total number of EBA-positive microvessel profiles. Welch’s t-test: t = 5.86, df = 1789, p-value < 0.001

Group	N	Mean surface area	SE
Control	1515	26.68	0.98
Experimental	1274	8.58	0.33

**Table 2:** Mean surface areas of EBA-positive microvessels in the cerebellum. N: total number of microvessel profiles. Welch’s t-test: t=73.40, df=1337, p-value < 0.001

Group	N	Mean surface area	SE
Control	1100	11.11	0.5
Experimental	551	7.51	0.4

## Discussion

The purpose of the present study was to apply the computerized image analysis using the Olympus cellSens software to determine changes in EBA-immunoreactive microvessels in the rat forebrain and cerebellum more objectively and accurately from standardized microscopic fields following intraperitoneal injection of cadmium. In this study, capillaries and venules and their collaterals of varying lengths were labeled by the ant-EBA in all brain sections examined (Figures 2, 6). There were no apparent within-group differences in the caudal-rostral spatial distribution of the EBA-immunoreactive vasculature. An interesting observation from this study is the marked difference in the density of EBA-positive microvessels in the forebrain compared to the cerebellum in control rats (26.68 mµ^2^ and 11.11 mµ^2^, respectively). The specific functions of EBA in the CNS or the significance of these density differences are unclear but may reflect differences in metabolic needs between the two CNS regions or differential EBA expression accompanying ongoing angiogenesis and maturation of the nascent BBB-competent normal rat microvessels. Another noteworthy observation from this study is that in cadmium-exposed rats, density decreased to 7.51 mµ^2^ and 8.58 mµ^2^ in the forebrain, respectively. This represents a 32.4% decrease in the cerebellum compared to 67.8% in the forebrain of EBA-positive microvessels. These differences are significant (Figure 7) and appear to correlate with the histological observations (Figures 3, 4). The mechanisms underlying these cadmium-associated decreases in EBA-positive microvessels are currently unknown and warrant future investigation. A review of the literature indicates that most clinical manifestations of cadmium neurotoxicity are related to lesions in the cerebral neocortex, such as ADHD, dyslexia, mental retardation, neurobehavioral, and other cognitive disabilities [30,33,93]. The higher percentage change in the forebrain EBA-positive microvessels appears to correlate with the manifestation of cerebral symptoms commonly seen in cadmium neurotoxicity, although cadmium-related histopathological changes have been reported not only in the forebrain but also in the cerebellum and other brain regions in the rat [84,94] and swine [95] models of cadmium neurotoxicity. To further confirm that the measured cadmium-induced microvascular differences are due to alteration in EBA expression and not due to a general reduction in vascular endothelial density, we employed an antibody directed against endoglin (CD105) as a general endothelial marker. Endoglin is a 180 kDa transmembrane protein and is a used marker for studying active angiogenesis such as in brain development, tumors, or demyelinating CNS pathologies [96-97]. Immunofluorescent staining demonstrated that the EBA and CD105 are very different epitopes. While the anti-EBA is negative in peripheral tissues, the anti-CD105 strongly reacted with blood vessels at these anatomical locations (Figures 5E, 5F). Moreover, the anti-EBA immunoreactivity was restricted to microvessels within the brain neuropil and was completely negative in fenestrated endothelial cells in the kidney and choroid plexus (Figures 5B, 5E). By contrast, anti-CD105 strongly labeled endothelial cells within the brain and choroid plexus, apparently with equal efficiency (Figures 5C, 5F). Taken together, the above findings, along with quantitative changes reported, here suggest a true reflection of alterations in microvessel content of EBA and could not have been due to general changes in vasculature density. It also demonstrates that CD105 and EBA are two very different epitopes. Results from our current study suggest that EBA immunoreactivity provides a specific and accurately measurable indicator of cadmium toxicity on the developing, nascent BBB in the rat CNS. Many previous studies have documented the sensitivity of EBA-immunoreactivity for observational, qualitative evaluation of BBB involvement in infectious and non-infectious CNS diseases. In an investigation using Wistar rats that were injected with Clostridium perfringens prototoxin, Zhu et al. [79] found an inverse correlation between EBA immunoreactivity and BBB disruption as shown by increased permeability to endogenous albumin detected immunohistochemically both at the light and electron microscopic levels. Many authors have previously employed subjective and semi-quantitative methods to evaluate temporal-spatial dispositions of EBA immunoreactivity under normal and pathological conditions [75,77,80,82].

The introduction of a variety of computerized image analysis protocols has facilitated the performance of unbiased, accurate, and efficient quantitation of profiles in biological structures [98-99]. These methods have been extended to the evaluation of EBA under normal and pathological situations. For example, Sibbons and others [100] applied Cavalieri’s stereological principle of template point counting on fresh brain slices together with EBA immunohistochemistry on thick vibratome brain sections to determine age-related changes in total surface area of BBB-competent microvessels per unit volume of brain tissue in normal rat neocortex. A study by Jin et al. [101] utilized an Image-Proplus 5.0 medical imaging analysis software was used to measure the integrated optical density (IOD) as an indicator of EBA-immunoreactivity in the rat's parietal cortex following targeted irradiation. Another study by El-Salhy et al. [102] employed the Olympus cellSens like that described in our present study to compute the total number of endocrine cells per unit area of tissue in ileal biopsies from patients with irritable bowel syndrome. The authors used manual counting by mouse-pointing and clicking on profiles of interest and drawing the areas containing those profiles on the computer screen. More recently, Youssef et al. [103] utilized the Olympus cellSense Dimension Software with manual delineation of profiles of interest like the study by El-Salhy et al. [102], to determine the area of the dentate gyrus and granule cell layer in thick sections from the developing mouse brain after exposure to stress. By contrast, we captured all profiles of interest by setting a global threshold and applying this to all fields to automatically generate the numbers and surface areas of the BBB-competent microvessels rapidly and unbiasedly in each FOV. Other investigators have applied similar quantitative computer-assisted image analysis to determine the number and areal fraction of EBA-immunopositive microvessel profiles within defined microscopic fields in a rat model of traumatic brain injury [78]. Taken together, these studies indicate that the EBA is a sensitive and reliable indicator for evaluating BBB involvement in the pathogenesis of rat models of CNS disorders.

Despite the use of gestational, newborn, and adult rats as valid model systems for studying the neurotoxic effects of cadmium [104-109], the possible alterations in EBA in the developing rat brain as a result of cadmium exposure have not been widely studied. Rodents, including rats and mice, have been extensively used in studies to understand the pathophysiologic mechanisms of environmentally induced CNS pathologies such as cadmium intoxication and other disorders in humans. To reliably extrapolate findings from such studies, it is important to recognize the prenatal and postnatal species differences in brain development. In terrestrial vertebrates, including rats and humans, CNS development begins with the formation of the neuroectoderm-derived neural tube, which then progresses through three primary and five secondary brain vesicles that ultimately differentiate into forebrain, midbrain, cerebellum, medulla oblongata, and spinal cord. These stages occur at different rates in rats and humans. Details of the comparative developmental neuroanatomy and the identification of key landmarks when the developing rat and human brains appear to be more susceptible to the impact of pathogenic agents, including cadmium exposure, have been well described in the literature. A review of these studies indicates that most of the key developmental processes, including changes in brain volume, weight, gross structure, formation of synaptic contacts, gliovascular unit (BBB), myelination, and neurotransmission activities, continue through the postnatal periods in both rat and man, though at different time scales. These studies also indicate that the rat brain at postnatal day 10 is developmentally equivalent to the human brain milestone of three months (first trimester) intrauterine life. Further, five weeks postnatal rat brain is developmentally estimated to correspond to a human brain at age 2-4 years (under-five-year-old). At these time points, the rat and human CNS and its BBB gliovascular structures are immature compared to similar adult structures. It is conceivable, therefore, that both rat and human BBB at these early stages are more susceptible to cadmium exposure in both acute, subacute, and chronic settings compared to adult brains [110,111].

Here we have investigated a single timepoint evaluation of the impact of cadmium exposure during the neonatal period. To address this limitation, additional longitudinal studies are underway whereby we will expose the developing rat brain from gestation through birth to post-weaning, collecting tissue at each time point post-partum. This will allow us to more closely mimic human exposure to the metal for downstream analysis of postnatal EBA recovery or otherwise from the impact of chronic cadmium exposure *in utero*.

## Conclusions

The results from the present study suggest that EBA could serve as a potential tool for accurately and reproducibly analyzing BBB alterations following exposure to cadmium in the rat model system. The cellSens computer-assisted quantitative methodology described in this study could serve as a foundational reference for future mechanistic investigations of the pathophysiologic mechanisms of cadmium neurotoxicity in this model of developing, in situ rat BBB model system. 
